# Single Circulating Fetal Trophoblastic Cells Eligible for Non Invasive Prenatal Diagnosis: the Exception Rather than the Rule

**DOI:** 10.1038/s41598-020-66923-9

**Published:** 2020-06-17

**Authors:** Laure Cayrefourcq, Marie-Claire Vincent, Sandra Pierredon, Céline Moutou, Marion Imbert-Bouteille, Emmanuelle Haquet, Jacques Puechberty, Marjolaine Willems, Cathy Liautard-Haag, Nicolas Molinari, Cécile Zordan, Virginie Dorian, Caroline Rooryck-Thambo, Cyril Goizet, Annabelle Chaussenot, Cécile Rouzier, Amandine Boureau-Wirth, Laetitia Monteil, Patrick Calvas, Claire Miry, Romain Favre, Yuliya Petrov, Philippe Khau Van Kien, Elsa Le Boette, Mélanie Fradin, Catherine Alix-Panabières, Claire Guissart

**Affiliations:** 1 Laboratoire de Génétique de Maladies Rares, Institut Universitaire de Recherche Clinique, EA7402 Université de Montpellier, CHU Montpellier, Montpellier, France; 2 Laboratory of Rare Human Circulating Cells (LCCRH), University Medical Center of Montpellier, Montpellier, France; 3 Laboratoire de Diagnostic Préimplantatoire, Hôpitaux Universitaires de Strasbourg, site du CMCO, Strasbourg, France; 4 Département de Génétique Médicale, Maladies Rares et Médecine Personnalisée, Centre de Référence Anomalies du Développement et Syndromes Malformatifs, Université de Montpellier, CHU de Montpellier, Montpellier, France; 5 DIM, CHU Montpellier - Hôpital la Colombière, Montpellier, France; 6 Service de Génétique Médicale, Groupe Hospitalier Pellegrin, CHU de Bordeaux, Bordeaux, France; 7 Service de Génétique Médicale, Centre de Référence des Maladies Mitochondriales, Hôpital de l’Archet 2, Nice, France; 8 Service de Génétique Médicale, CHU de Toulouse, Toulouse, France; 9 Department of Maternal Fetal Medicine, Strasbourg University Hospital, Strasbourg, France; 10 Laboratoire de Cytologie Clinique et Cytogénétique, CHU de Nîmes, Nîmes, France; 11 Service de Génétique Médicale, Centre Hospitalier de Saint Brieuc, Saint-Brieuc, France

**Keywords:** Biotechnology, Cell biology, Genetics, Molecular biology, Diseases, Health care, Medical research, Molecular medicine

## Abstract

Non-Invasive Prenatal Diagnosis (NIPD), based on the analysis of circulating cell-free fetal DNA (cff-DNA), is successfully implemented for an increasing number of monogenic diseases. However, technical issues related to cff-DNA characteristics remain, and not all mutations can be screened with this method, particularly triplet expansion mutations that frequently concern prenatal diagnosis requests. The objective of this study was to develop an approach to isolate and analyze Circulating Trophoblastic Fetal Cells (CFTCs) for NIPD of monogenic diseases caused by triplet repeat expansion or point mutations. We developed a method for CFTC isolation based on DEPArray sorting and used Huntington’s disease as the clinical model for CFTC-based NIPD. Then, we investigated whether CFTC isolation and Whole Genome Amplification (WGA) could be used for NIPD in couples at risk of transmitting different monogenic diseases. Our data show that the allele drop-out rate was 3-fold higher in CFTCs than in maternal cells processed in the same way. Moreover, we give new insights into CFTCs by compiling data obtained by extensive molecular testing by microsatellite multiplex PCR genotyping and by WGA followed by mini-exome sequencing. CFTCs appear to be often characterized by a random state of genomic degradation.

## Introduction

Non-Invasive prenatal diagnosis (NIPD) of monogenic diseases, based on the analysis of circulating cell-free fetal DNA (cff-DNA)^1–3^, is a safer alternative to invasive prenatal testing methods (amniocentesis and choriocentesis) that entail a significant risk of miscarriage (0.5–1%)^4^. However, technical issues related to cff-DNA characteristics remain^5^. Consequently, not all mutations can be investigated with this approach, particularly triplet expansion mutations that concern rare and incurable diseases (e.g., Steinert myotonic dystrophy, Huntington’s disease, fragile X syndrome, spinocerebellar ataxia type 1, 2, 3). Indeed, the strong fragmentation and the short size of cff-DNA (143 bp) do not allow the direct detection of these mutations. However, this group of pathologies represents a frequent prenatal diagnosis indication. An alternative approach is to perform analysis on circulating fetal trophoblastic cells (CFTCs) isolated from maternal blood.

Several methods have been used to isolate CFTCs from maternal blood^6–8^. However, to date, no CFTC-based test is reliable enough for routine application as NIPD for monogenic disease. Recently, new enrichment and detection systems have been optimized for circulating tumor cells (CTCs) as a liquid biopsy of solid cancer^9,10^. Some of these new technologies could be easily applied to CFTC enrichment, detection and characterization. Indeed, these two populations of circulating cells are both very rare in the bloodstream and require the same technical enrichment steps to distinguish them from the surrounding normal blood cells (e.g., leukocytes).

The objective of this study was to develop an approach for NIPD of monogenic diseases caused by point mutations or triplet repeat expansions based on the isolation of single CFTCs from maternal peripheral blood. We evaluated the suitability of enrichment technologies currently used for CTCs using Huntington’s disease (HD) as a clinical application model. Then, we analyzed the CFTC characteristics by compiling data obtained with different molecular analysis methods: microsatellite multiplex PCR genotyping for HD and Whole Genome Amplification (WGA) followed by mini-exome sequencing for point mutation detection.

## Materials and Methods

### Artificial maternal blood preparation

For the development and validation phase of the CFTC isolation method, two different materials were used to mimick maternal blood samples: (i) peripheral blood samples from healthy donors provided by the Établissement Français du Sang; and (ii) primary trophoblastic cells obtained from the Cytogenetic department, Montpellier Hospital, France, and the HTR-8/SVneo cell line (ATCC CRL-3271). Blood samples were collected in different vaccum tubes, EDTA, TransFix (TransFix Cytomark), and Streck (Cell-Free DNA BCT Streck), and spiked with different amounts of trophoblastic cells, labelled with calcein (C3099-Molecular Probes, Invitrogen), to evalute the recovery rate. Cell culture conditions are described in the Supplementary Methods.

### Participants and blood sample collection

The study participants were couples at risk of transmitting a monogenic disease to their fetus. They were recruited through two clinical trials: DIACCIMEX (*ClinicalTrials.gov*, NCT 03087526) and DIAFEXOME (*ClinicalTrials.gov*, NCT 03743948). Sample collection for research was approved by the Sud Est V Research Ethics Committee (Personal Protection Committee, CPP), on April 18, 2017, for couples at risk of transmitting HD (DIACCIMEX), and by the Ile de France VI CPP, on October 9, 2018, for couples at risk of transmitting monogenic diseases covered by the TruSight One expanded mini-exome sequencing kit^11^ (DIAFEXOME). Informed consent was obtained from all participants. All methods of the study were carried out in accordance with relevant guidelines and regulations.

In total, 16 couples were included (n = 7 at risk of transmitting HD; n = 9 at risk of transmitting a monogenic disease caused by point mutations). Pregnant women underwent prenatal diagnosis (gold standard) by amniocentesis or choriocentesis between week 10 and 16 of gestation, after blood sampling for NIPD. Couples were included in the study at one of the participating medical genetic centers during a genetic counseling consultation. During this visit, blood samples were collected in Streck (3 × 10 mL) and EDTA (1 × 5 mL) tubes for the pregnant women, and in EDTA tubes (1 × 5 mL) for the future fathers. Blood samples were processed in less than 24 h (Streck tubes) and less than 7 days (EDTA tubes). Blood samples collected in Streck tubes were used for CFTC isolation and analysis. ﻿Genomic DNA was extracted from the blood collected in EDTA tubes with the FlexiGen DNA kit (Qiagen), according to the manufacturer’s protocol, for genotyping.

### CFTC Isolation method

CFTCs are isolated using a method that combines enrichment and multicolor staining for CFTC identification, and isolation techniques. Two different techniques were evaluated for CFTC enrichment from peripheral blood: the Parsortix system and the RosetteSep technology with the CTC enrichment kit (15167-Stemcell Technologies), according to the manufacturers’ instruction. The Parsortix system is a size-based positive enrichment method (using the cell physical properties to differentiate CFTCs from leukocytes), while the RosetteSep technology is a negative enrichment technique based on antibodies that target unwanted cells to remove them from whole blood (using the cell biological properties to differentiate CFTCs from leukocytes). The detection step is based on the labeling of the enriched fraction with a cocktail of fluorescent antibodies to differentiate CFTCs from the remaining normal leukocytes after cell fixation and permeabilization. Specifically, the anti-CD105-PE (Miltenyi), -PanCK-FITC (against cytokeratin 8, 18, and 19) (Miltenyi), and -βhCG-FITC (Biotechne) antibodies were used for CFTC detection, and the anti-CD45-APC (Miltenyi) antibody to label residual blood cells. Finally, CFTCs were identified and sorted using the DEPArray system (Supplemental Method), a dielectrophoresis technology allowing visual selection and single cell sorting for, downstream analysis as well as single residual leukocytes to evaluate the cell quality of the sample. A genetic confirmation of the origin of each isolated cell was then performed by the multiplex PCR protocol described in the following paragraph.

### Molecular diagnosis of huntington’s disease

The molecular diagnosis protocol used in this study was adapted from the preimplantation genetic diagnosis method for HD described by Moutou *et al*. in 2004^12^ for single-cell analysis^13^. This protocol is based on two approaches: (i) direct testing of the (CAG)n triplet repeat, and (ii) linkage analysis using one or more intragenic or flanking microsatellite markers, in addition to the direct approach (See Supplementary Methods, Fig. S1, Tables S1 and S2).

The allele drop-out (ADO) rate was estimated in all samples for which a PCR signal was obtained at least at one tested locus, as follows:

$${\rm{ADO\; =\; (expected\; alleles\; -\; detected\; alleles)/(expected\; alleles)}}$$

**Figure 1:**
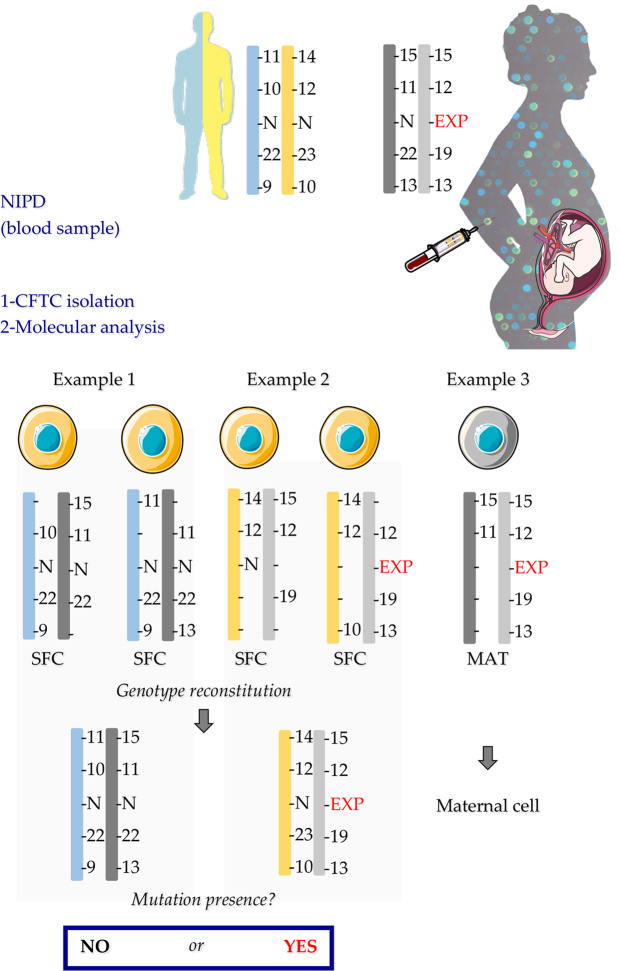
Genotype result interpretation. In example 1 and 2, the presence of the paternal-specific alleles 10 and 14 allows concluding that these two single cells are fetal cells. Example 3 illustrates the identification of a maternal cell on the basis of its genotype. EXP: expansion of triplet repeats; MAT: maternal cell; N: normal triplet repeat size; NIPD: non-invasive prenatal diagnosis; SFC: Single Fetal Cell.

Genotypes were interpreted in two steps. First, single fetal cells were distinguished from maternal cells on the basis of the presence of paternal-specific alleles (Fig. 1). Then, the parental haplotypes were deduced from the CFTC genotype analysis.

### Molecular diagnosis of monogenic diseases by NGS

For the other monogenic diseases tested using CFTCs, first, genomic DNA from single cells was amplified by WGA with the REPLI-g Single Cell Kit (Qiagen), according to the manufacturer’s instruction. After confirmation of the fetal origin of the WGA products using a set of informative short tandem repeat (STR) markers, mini-exome sequencing was performed by exon capture with the TruSight One Sequencing Panel Kit, as described previously^14^, on an Illumina NextSeq sequencer.

TruSight One Expanded is designed for genomic analysis of the coding regions of 6,699 genes with clinical phenotypes.

To evaluate the single-cell sequencing quality, the mini-exome coverage at 30X depth was compared between single cells and the 23 independent DNA samples without amplification, sequenced in the same run (i.e., the reference; considered to have 100% coverage).

Then, the sequences of the CFTC samples were compared with the relevant maternal and paternal sequences to determine the inheritance of specific maternal and paternal variants in order to detect potential amplification biases. Finally, the presence of the known disease-causing mutation was determined by visual inspection of the mapped reads using Integrative Genomics Viewer (IGV).

### NIPD interpretation and comparison with the invasive pre-natal diagnosis results

The NIPD and invasive pre-natal diagnosis results (expressed as affected or non-affected fetus) were compared in blind.

## Results

### Method for CFTC isolation

#### Selection of markers for CFTC identification

To select markers that could be used to identify CFTCs in peripheral blood samples, we first evaluated several antibodies against previously used markers (Supplementary Table S3) in the HTR-8/SVneo trophoblastic cell line and in primary trophoblasts. Both cell types were positive for CD105, βhCG, EGFR, vimentin and CD44, and negative for HLAG, CD141, CD146 and CD45. We did not detect cytokeratin 7 (CK7) expression in both cell types, whereas CK8, 18, and 19 (PanCK antibody) were strongly expressed in HTR-8/SVneo cells and in 30% of primary trophoblasts. Therefore, we selected the positive markers CD105, βhCG and PanCK for CFTC identification and CD45 for leukocyte exclusion. We did not include EGFR because this marker is also expressed in erythroblastic fetal cells^15,16^.

#### CFTC Enrichment in spiking experiments

Based on literature data^17^, we first decided to use the Parsortix system with 6.5 µm cartridges, a size-based enrichment technique to isolate CFTC from maternal blood. To evaluate its performance we spiked blood from healthy donors with HTR-8/SVneo cells, to mimick maternal blood, and cells were pre-labeled with calcein to measure the recovery rate at each step of the process using fluorescent microscopy (Supplementary Fig. S2). We also compared three different vacuum blood tubes (EDTA, TransFix, and Streck) to find the best pre-analytical conditions.

With blood collected in EDTA tubes, we obtained 50% of capture in the cartridges and 34% of recovery after cell harvesting from the cartridge (Table 1), in agreement with the data reported by the manufacturer. However, we lost most of the enriched cells during the labeling step. Indeed, cells in EDTA tubes remained viable during the enrichment step and therefore they were more likely to be damaged by any additional handling steps. Conversely, the TransFix and Streck tubes contain a fixative that keeps cells intact for few days. In agreement, 97% and 48% of HTR-8/SVneo cells were captured in the cartridge, and 92% and 40% were recovered from samples collected in TransFix and Streck tubes, respectively (Table 1). Samples in TransFix tubes displayed the best recovery rate, but the number of remaining white blood cells (around 200,000 for a blood sample processed within 24 h) was too high to be combined with the DEPArray system for pure single cell isolation. Indeed, HTR-8/SVneo cells could be detected but not isolated as pure single cells because there were always several leukocytes (from 2 to 10) in the same DEPcage. Therefore, the final recovery rate with the DEPArray system remained very low even for samples collected in TransFix tubes.

**Table 1:** Recovery rate of each step of the Parsortix and RosetteSep enrichment methods.

	Parsortix				
	Spiking	Capture	Recovery	Labeling	DEPArray
EDTA (n = 8)	121	60.5	41	11.5	2
		50%	34%	10%	2%
Transfix (n = 8)	89	86	82	56	13,5
		97%	92%	63%	15%
Streck (n = 2)	111,5	54	45	35,5	7
		48%	40%	32%	6%
	**RosetteSep**				
	**Spiking**		**Recovery**	**Labeling**	**DEPArray**
EDTA (n = 10)	132		100	86	18
			76%	65%	14%
Transfix (n = 2)	105		not applicable		
Streck (n = 6)	87		81	74	25
			93%	85%	29%

Next, we evaluated the RosetteSep enrichment technique that is based on leukocyte depletion. Like for the Parsortix system, we mimicked maternal blood by spiking blood from healthy donors with HTR-8/SVneo cells labeled with calcein. With this system, we obtained recovery rates of 76% and 93% (Table 1) using EDTA and Streck tubes, respectively. TransFix tubes could not be used with this technique because some components of the fixative solution prevent blood cells from getting through the density gradient medium. The final recovery rates in the DEPArray system were 14% and 29% with samples collected in EDTA and Streck tubes, respectively.

Moreover, to validate the whole process, we confirmed that the isolated single cells recovered from the DEPArray were HTR-8/SVneo using the Huntington’s Disease Multiplex PCR (Supplementary Fig. S3).

On the basis of these results, we decided to collect blood samples in Streck tubes, and to use the RosetteSep as enrichment method coupled with DEPArray detection and isolation.

### Clinical results

#### Triplet repeat expansion analysis: huntington’s disease multiplex PCR

We then used Streck tubes and the RosetteSep enrichment method to isolate CFTCs for NIPD from blood samples of seven pregnant women from couples at risk of transmitting HD to their fetus (blood sampled at 11–13 weeks of gestation).

The DEPArray system allowed the selection and isolation of a median of 15 (range 5–24) potential CFTCs per patients, on the basis of their phenotype (i.e., CD105- and/or PanCK/βhCG-positive and CD45-negative). In total, we could genotype 107 cells by multiplex PCR for HD. Table 2 gives an overview of the families’ characteristics and the single-cell analysis results, according to cell classification criteria of the genotyping results (Supplementary Table S4).

**Table 2:** Families’ characteristics and results of single-cell testing for Huntington’s disease.

Family	A	B	C	D	E	F	G	Total
Weeks of gestation + day	11 + 6	13 + 5	12 + 4	13	12	12 + 1	13 + 1	
Expansion carrier	Mother	Mother	Father	Mother	Mother	Father	Father	
Uninterpretable cell	11	18	5	9	16	6	5	70
Maternal cell	2	0	0	8	0	14	0	24
Inconclusive cell (maternal or fetal)	5	0	0	0	0	3	0	8
Fetal cell	1	1	0	2	0	1	0	5
NIPD result	n/c^a^	n/c	n/c	No mutation	n/c	n/c	n/c	1

^a^n/c: not conclusive.

Finally, we confirmed that only five of the isolated cells were CFTCs from four pregnant women (Families A, B, D, and F) (Table 2). Among these five CFTCs, 3/5 were CD105-positive, 1/5 was CD105- and PanCK/βhCG-positive, and 1/5 was only PanCK/βhCG-positive. The other isolated cells were maternal cells (n = 24) or with uninterpretable results (n = 70). In this last category, 42 cells had no parental-specific allele amplification, 17 presented artefactual pics and/or ≤1 maternal allele, 9 presented a contamination signal, and 2 corresponded to a mix of a maternal cell and a fetal cell. Moreover, 8 cells were in the inconclusive category that corresponds to genotypes with at least two alleles detected without the possibility to categorize the cell as maternal or fetal because of ADO (i.e., absence of paternal-specific allele and absence of two specific-maternal alleles at an informative marker locus).

**Figure 2:**
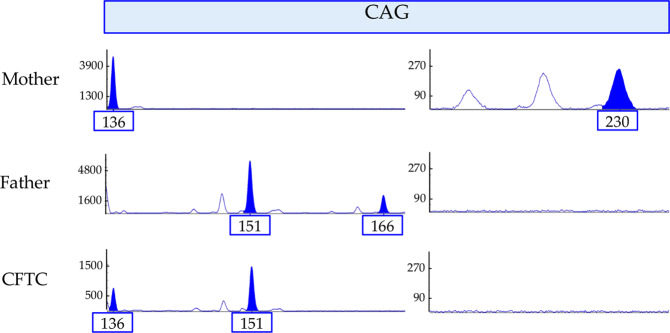
NIPD result for Family D. At the CAG repeat locus region, the mother carries one normal allele (136) and one expanded allele (230) that corresponds to a 44 repeats expansion mutation. The father carries two normal alleles (151 and 166). The fetus inherited the maternal normal 136 allele and the paternal normal 151 allele.

Only one of the five CFTCs tested led to a conclusive NIPD. In family D, the fetus inherited the two parental normal alleles in the triplet repeat region (Fig. 2). This conclusion was in agreement with the classic invasive prenatal diagnosis result.

Because of ADO, we could not conclude about the mutation inheritance in the other three families. Indeed, ADO rate (based on the number of detected and expected alleles) was significantly higher in single CFTCs than in maternal cells (χ^2^ = 67.9, p-value <0.001) (Table 3).

**Table 3:** ADO rate in the single maternal cells and CFTCs analyzed by multiplex PCR for Huntington’s disease.

	Maternal cells	CFTCs
No. of cells	24	5
No. of detected alleles	274	18
No. of expected alleles	356	68
ADO (%)^a^	23	74

^a^χ^2^ = 67.9, p-value < 0.001.

#### Mini-exome sequencing of CFTCs

In the second part of the project, we collected blood samples from nine couples at risk of transmitting a monogenic disease to their fetus to determine whether mini-exome sequencing of single CFTCs after WGA could be used as single testing method for a large panel of genes that cause monogenic diseases (Supplementary Table S5). Due to a technical issue, we lost the cells for three families (H, I and P) in the last DEPArray step when the selected single cells are moved from the cage to the tube. For the other six couples, we selected and isolated a median of 9 potential CFTCs per patient (range 2–30) (Supplementary Table S5). Among them, we identified two CFTCs (Families J, and M) by STR analysis. These two CFTC were CD105/PanCK/βhCG-positive (Family J) and CD105-positive (Family M).

**Figure 3:**
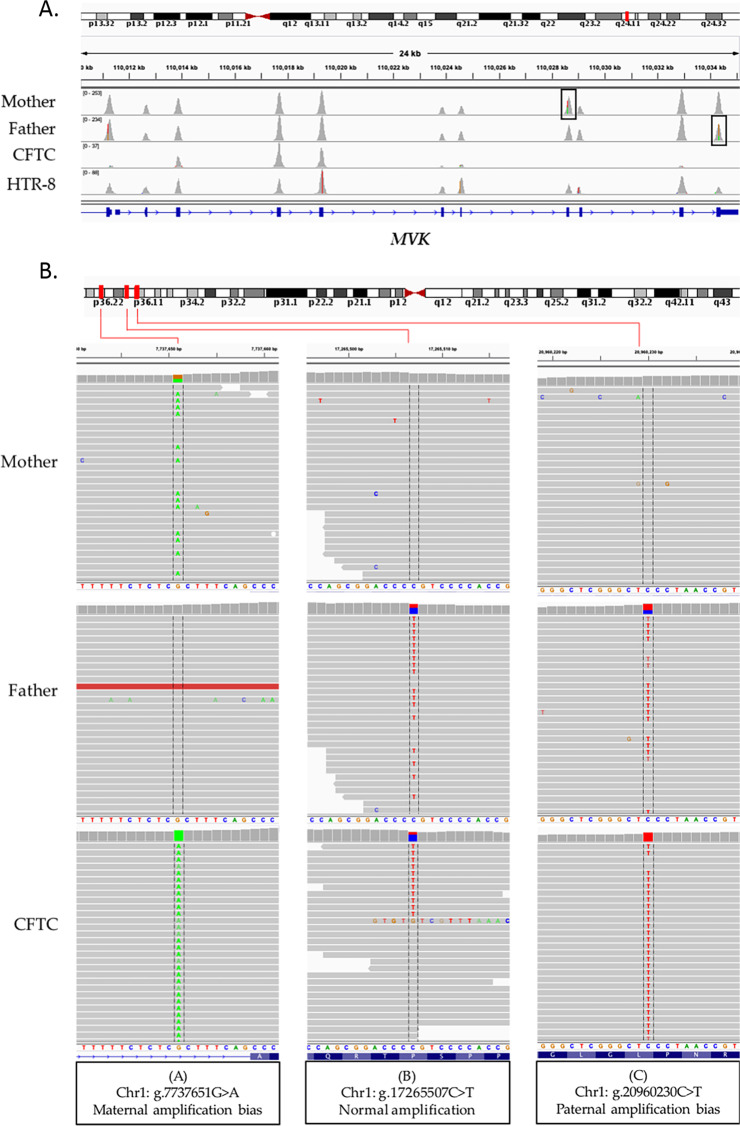
(**A**) IGV visualization of the sequence coverage in the *MVK* gene on chromosome 12 in the couple at risk of transmitting mevalonic aciduria to their fetus (Family J) and in a single HTR-8/SVneo cell from a different sequencing run. The maternal and paternal mutations targeted in this analysis are located in exon 8 and 11 (highlighted by black frames), respectively. (**B**) Amplification bias characterization in a single CFTC (Family J). IGV visualization of sequence alignments in three regions of chromosome 1 showing: (**A**) Abnormal amplification of a maternal allele (the maternal heterozygous variant is homozygous in the CFTC), (**B**) Normal amplification (both wild type and paternal variant are found in CFTC), and (**C**) Abnormal amplification of a paternal allele.

We first evaluated the performance of the single-cell sequencing protocol using single HTR-8/SVneo cells. Compared with the genomic DNA samples (100% coverage), the targeted base coverage (30X depth) was 59% for single HTR-8/SVneo cells. On the other hand, mini-exome sequencing of the confirmed isolated CFTCs allowed a targeted base coverage at 30X of 23% (family J) and 4% (family M), indicating a very poor coverage. Consequently, we could not reach any conclusion for Family J at risk of transmitting mevalonic aciduria to their fetus (the mother and the father harbored two different missense mutations in the *MVK* gene) (Supplementary Table S5). Indeed, mini-exome analysis of the confirmed CFTC showed that the exons harboring the parental missense mutations were not covered by any reads, suggesting locus drop-out during WGA (Fig. 3A). Only exons 3, 4 and 5 had sufficient coverage. On the other hand, WGA followed by mini-exome sequencing of a single HTR-8/SVneo cell allowed obtaining full coverage (Fig. 3A).

To investigate the risk of false negative and false positive results, we looked for specific parental polymorphisms (mother heterozygous and absent in the father, and *vice versa*) in regions with more than 30X coverage in the CFTC sample from Family J. Unfortunately, we found several amplification biases randomly distributed along genomic regions. Figure 3B shows examples of amplification biases identified in chromosome 1.

In family M (risk of hereditary distal motor neuropathy type VI due to maternal and paternal mutations in the *IGHMBP2* gene) (Supplementary Table S5), none of the *IGHMBP2* exons was covered by mini-exome sequencing. This result was not surprising given the very low coverage (4%).

## Discussion

As cff-DNA analysis for NIPD still presents technical issues for some mutations^5,18^, particularly triplet expansion mutations (e.g., HD), we wanted to develop an alternative approach using CFTCs isolated from maternal blood. Theoretically, CFTCs represent the ideal material for NIPD because total non-fragmented fetal DNA could be available for downstream analysis without maternal contamination. However, these cells are very rare in the bloodstream, and therefore extremely sophisticated technologies are needed for their isolation and analysis, as previously reported for CTCs in liquid biopsy. During the last decade, tremendous efforts have been made to overcome the technological difficulties of CTC analysis, and now several devices, methods and protocols are available for the enrichment, detection, isolation and characterization of these rare cells in blood^10^. Most of these technologies should be applicable to CFTCs.

Several methods have been developed to isolate CFTCs from maternal blood^6,7,19–23^. Among them, the ISET system, initially used for CTC analysis, is the only CFTC approach successfully used in monogenic diseases caused by point mutations^6^. However, this technology has not been translated in the clinical practice. The other published methods focus on the cytogenetic analysis of CFTCs for the detection of large chromosomal rearrangements. The latest patented method, a cell-based non-invasive prenatal test for copy number analysis, was tested in five pregnant women^23^. However, this protocol, which uses pools of 2–7 CFTCs per analysis and high DAPI concentration, was not tested for point mutation screening.

Here, we tested two different technologies for CFTC enrichment from blood samples (Parsortix and RosetteSep) combined with the DEPArray system, a high-technology method to detect and enrich pure single cells^24^. We observed that the efficiency of the whole procedure relied on the type of tube used for blood sampling, and confirmed that the pre-analytical steps are crucial. To date even if the Parsortix gave good results in term of enrichment of CFTCs, its combination with the DEPArray is not appropriate for single cell isolation. We obtained the best results (final recovery rate of about 30%) using the RosetteSep technology with blood collected in Streck tubes. We then used this method to isolate CFTCs from blood samples from 16 pregnant women at risk of transmitting a monogenic disease to their fetus.

According to the literature, we expected a minimum of 60 CFTCs per 30 mL of maternal blood sample^4,5^. Thus, considering the 30% isolation yield of our approach, we should have detected about 18 CFTCs per sample. We obtained a mean of 12 potential CFTCs per patients (range 2–30). Unfortunately, among all the isolated cells, we confirmed their CFTC nature by molecular analysis only in few cases (i.e., 1–2 CFTCs in 6 of the 16 blood samples used in this study). Specifically, in the couples at risk for transmitting HD to their fetus, we confirmed the CFTC nature of the isolated cells by detecting paternal-specific alleles by multiplex PCR. In this group, the ADO rate in single maternal cells (23%) was slightly higher than the values usually described for single-cell analysis. Moutou *et al*. estimated at about 11% the percentage of ADO in blastocysts with a similar PCR protocol^12^. Our result could be explained by the cell isolation protocol, particularly the fixation and permeabilization steps, that could have damaged the DNA and led to higher ADO rates. However, ADO rate in CFTCs was 3-fold higher (74%) than in maternal cells, although they underwent the same treatment. This suggests that CFTCs are more fragile than expected, probably because of apoptosis linked to their presence in blood (unnatural microenvironment), compared with maternal cells (mainly circulating leukocytes). Unlike CTCs in which the ability to spread is linked to their capacity to resist to apoptosis and anoikis^25–27^ in order to survive in other microenvironments, CFTCs might have ended in the maternal bloodstream precisely because they were undergoing apoptosis^28^.

This high ADO rate might explain the absence of diagnostic conclusion for 4 of the 5 confirmed single CFTCs isolated in the HD group. In addition, the absence of data on the grandparents and previous child’s genotypes for parental haplotype determination did not allow the indirect diagnosis. When designing the protocol, we thought that we could deduce the haplotype while performing the CFTC analysis, on the basis of the parents’ genotypes. However, this was not possible due to the high ADO rate and the low number of isolated cells.

These results suggest that CFTC DNA is most of the time damaged and that the molecular analysis would be complicated with any of the currently available molecular diagnostic methods. In agreement, WGA combined with mini-exome sequencing of CFTCs did not improve NIPD performance. WGA protocols increase the amount of DNA present and offer the possibility of using different downstream molecular biology protocols. However, they are sensitive to the DNA quality. Indeed, our results showed that the WGA approach did not overcome the lack of DNA quality, as indicated by the amplification biases randomly distributed in different genomic regions and the very low coverage obtained by mini-exome sequencing.

We chose the REPLI-g Single Cell Kit because a comparison study of five single-cell WGA kits^29^ showed that with this kit, coverage is higher with a comparatively low ADO rate in single primary human foreskin fibroblast cells^29^. Therefore, the low coverage observed in our study could be explained by an insufficient amount of WGA product available for the mini-exome library preparation. Indeed, the post-WGA DNA amount is difficult to estimate, because high molecular weight products can be generated by random primer-dimer production. Depending on the input DNA quality, the resulting DNA might be of lower quality (fragmented or damaged DNA), but the DNA amount would be similar, masked by the primer-dimer signal.

Altogether, the results obtained with different molecular tests (microsatellite multiplex PCR genotyping and WGA followed by mini-exome sequencing) indicate the CFTC DNA is often degraded. This partly explains the current inability to propose a routine NIPD protocol based on CFTC analysis for monogenic diseases caused by point mutations. It also emphasizes the importance of having not a single diagnostic cell but multiple diagnostic cells, recently recommended by Vossaert L *et al*. (2019) for CNV analysis^30^. Indeed, the analysis of each single CFTC is critical because first, the quality of these cells can vary substantially since some cells are in an apoptotic state involving genome-wide degradation. Secondly, in twin or higher-multiple pregnancies or the case of confined placental mosaicism, adequate genotyping is necessary to confirm the fetal origin of each cell and to potentially distinguish different genetic signatures.

In single cell-based NIPD, another limitation is the question whether fetal cells present in maternal blood from earlier pregnancies will interfere. It has been demonstrated that other types of circulating fetal cells, including lymphoid and myeloid fetal cells, are not cleared from blood rapidly after delivery, thus being potential source of false positive or false negative results^31^. Consequently, it is particularly important to raise great specificity in fetal trophoblastic cells isolation, which display a unique combination of markers^6^.

Our results show that our method, combination of negative enrichment and DEPArray sorting, although based on cutting-edge technology, is not reliable enough for NIPD where high specificity and efficient cell recovery are needed. This technique also presents the disadvantage of being a bit long, while prenatal investigations require short analysis times and little available material.

## Conclusion

CFTCs rarity and degradation caused by apoptosis are the two major limitations to the development of a robust NIPD protocol for microsatellite, dynamic mutation and point mutation analysis. Indirect methods based on relative dosage haplotyping of cff-DNA represent possible alternatives, but they are not suitable in the case of consanguinity, which is a frequent condition in prenatal diagnosis settings. Moreover, indirect methods still face several technical challenges and need complex bio-informatic analyses. Thus, an NIPD method based on CFTC for monogenic disease analysis is still needed.

## Supplementary information

Supplementary information.
