# Supplementary material for: Single Circulating Fetal Trophoblastic Cells Eligible for Non Invasive Prenatal Diagnosis: the Exception Rather than the Rule
Source: Sci Rep. 2020 Jun 17;10:9861. doi: 10.1038/s41598-020-66923-9 (PMC7300110; doi:10.1038/s41598-020-66923-9)

Supplementary Information

Single Circulating Fetal Trophoblastic Cells Eligible for Non Invasive Prenatal Diagnosis: the Exception Rather than the Rule.

Laure Cayrefourcq [2,12], Marie-Claire Vincent [1,12], Sandra Pierredon [1], Céline Moutou [3], Marion Imbert-Bouteille [1], Emmanuelle Haquet [4], Jacques Puechberty [4], Marjolaine Willems [4], Cathy Liautard-Haag [1], Nicolas Molinari [5], Cécile Zordan [6], Virginie Dorian [6], Caroline Rooryck-Thambo [6], Cyril Goizet [6], Annabelle Chaussenot [7], Cécile Rouzier [7], Amandine Boureau-Wirth [7], Laetitia Monteil [8], Patrick Calvas [8], Claire Miry [9], Romain Favre [9], Yuliya Petrov [10], Philippe Khau Van Kien [10], Elsa Le Boette [11], Mélanie Fradin [11], Catherine Alix-Panabières [2,13] and Claire Guissart [1,13,*]

[1] Laboratoire de Génétique de Maladies Rares, Institut Universitaire de Recherche Clinique, EA7402 Université de Montpellier, CHU Montpellier, Montpellier, France; claire.guissart@inserm.fr, sandra.pierredon@hotmail.fr, m-imbert-bouteille@chu-montpellier.fr, cathy.haag@live.com, marie-claire.vincent@inserm.fr

[2] Laboratory of Rare Human Circulating Cells (LCCRH), University Medical Center of Montpellier, Montpellier, France; l-cayrefourcq@chu-montpellier.fr, c-panabieres@chu-montpellier.fr

[3] Laboratoire de Diagnostic Préimplantatoire, Hôpitaux Universitaires de Strasbourg, site du CMCO, Strasbourg, France; celine.moutou@chru-strasbourg.fr

[4] Département de Génétique Médicale, Maladies Rares et Médecine Personnalisée, Centre de Référence Anomalies du Développement et Syndromes Malformatifs, Université de Montpellier, CHU de Montpellier, France; e-haquet@chu-montpellier.fr, j-puechberty@chu-montpellier.fr, m-willems@chu-montpellier.fr

[5] DIM, CHU Montpellier - Hôpital la Colombière, Montpellier, France; nicolas.molinari@inserm.fr

[6] Service de Génétique Médicale, Groupe Hospitalier Pellegrin, CHU de Bordeaux, Bordeaux, France; cecile.zordan@chu-bordeaux.fr, virginie.dorian@chu-bordeaux.fr; caroline.rooryck-thambo@chu-bordeaux.fr, cyril.goizet@chu-bordeaux.fr

[7] Service de Génétique Médicale, Centre de Référence des Maladies Mitochondriales, Hôpital de l'Archet 2, Nice, France; chaussenot.a@chu-nice.fr, rouzier.c@chu-nice.fr, boureau-wirth.a@chu-nice.fr

[8] Service de Génétique Médicale, CHU de Toulouse, Toulouse, France; monteil.l@chu-toulouse.fr, calvas.p@chu-toulouse.fr

[9] Department of Maternal Fetal Medicine, Strasbourg University Hospital, Strasbourg, France; claire.miry@chru-strasbourg.fr, romain.favre@chru-strasbourg.fr

[10] Laboratoire de Cytologie Clinique et Cytogénétique, CHU de Nîmes, Nîmes, France; yuliya.petrov@chu-nimes.fr, philippe.khauvankien@chu-nimes.fr

[11] Service de Génétique Médicale, Centre Hospitalier de Saint Brieuc, Saint-Brieuc, France; elsa.leboette@ch-stbrieuc.fr, melanie.fradin@chu-rennes.fr

[12] equally contributed to this work

[13] equally contributed to this work

Supplemental Methods

Cell Culture

Primary trophoblastic cells were maintained in Amniomedium/αMEM (50/50) (CCHCHR05-0U-Eurobio/22571-Gibco) supplemented with Fungizone (15290-Gibco), and HTR-8/SVneo cells were grown in RPMI 1640 medium (72400-Gibco) with 10% fetal bovine serum (CVFSF0001-Eurobio).

DEPArray procedure

DEPArray experiments were performed as recommended by the manufacturer. Briefly, after enrichement and labeling of the cells, the sample was washed twice in SB115 buffer (Silicon Biosystems – Menarini) as recommended for fixed cells. The sample was then concentrated in 12µl and loaded in the "S" part of DEPArray cartridge 2.0, followed by 830µl of SB155 buffer in the "B" part. Once prepared, the cartridge is loaded in the DEPArray system and the process started. First, the system automatically loads the sample in the main chamber of the cartridge and then asks the investigator to choose and adjust the settings for the scan of the main chamber. After the scan, the software offers the investigator different ways for the analysis. We always pick: 1) Selection of the event "in cage"; 2) View of the $CD105^{PE}$ positive events and selection of the putative CFTCs; 3) View of the PanCK / βhCG^{FITC} events and selection of the putative CFTCs. When all the CFTCs were selected the system moves them to the parking area where they were stocked before being sorted one by one in 0.2 ml tubes.

PCR protocol for Huntington's Disease Diagnosis

The PCR primer sequences are given in Supplemental Table 1. One primer of each pair was fluorescently labeled with the FAM, NED or HEX fluorochrome (Eurofin, MWG). The localization of the different markers is given in Supplemental Figure 1, and the PCR conditions are summarized in Supplemental Table 2. PCR products were analyzed on an ABI3130xl automated sequencer (Applied Biosystems, Warrington, UK; Genemapper software).

Supplemental Tables

Supplemental Table S1. Primer sequences.

Locus primer set	Direction-Primer sequence	Labeling	Fragment size range (bp)
D4S43 [1]	F-TCTTCCTTTTCTCTCTGGATGC<br>R-TGGAATTCTAGCTAGTTCAATAGGG	NED	88-100
D4S127.2	F-CCATTTTGCACTACGGAAAGGAGAAG<br>R-GTCCCTTGCATGCCCTGGCT	HEX	120-147
TG23P [1]	F-GTAGCCGAGATCCTGTCACTGT<br>R-GAAATTTGATTTTGGGAAGCCTATC	HEX	200-240
(CAG)n	F-ATGGCGACCCTGGAAAAGCTGATGA<br>R-GGCGGCTGAGGAAGCTGAGGA	FAM	154 for 16 CAG
I1CAHD.2	F-TATGCCACTACACTACAACCTGGGC<br>R-CACTTTGACAATACCACATGCTGGT	HEX	140-180
AC301 [1]	F-CAAGGATATGAAATGAATATTAGCTG<br>R-GTTCCATCTCCCTCTCCACTCC	HEX	280-330
D4S412	F-ACTACCGCCAGGCACTGGTAAGC<br>R-TAAGATATGAAAACCTAAGGGATAAAGG	NED	220-260
GT752D [1]	F-TTCCTGCCCACCTCCCTGAAG<br>R-CCTTCCCACTCTGACTTGTGC	FAM	120-135

[1] Markers not included in the protocol described by Moutou *et al.* 2004 (12). These additional primer sets were designed to improve the informativity within the couples.

Supplemental Table S2. PCR conditions.

Locus-primer set (pmol per reaction) [a]	Reagents	Thermocycling conditions
D4S43 (5 pmol)		
D4S127.2 (5 pmol)		95°C 15 min
D4S412 (5 pmol)		
GT752D (5 pmol)	Qiagen Master mix (1X)	[96°C 30s, 59°C 90s, 72°C 90s] x10
TG23P (10 pmol)	Q solution (1X)	[94°C 30s, 59°C 90s, 72°C 90s] x37
(CAG)n (10 pmol)		72°C 10 min
I1CAHD.2 (10 pmol)		
AC301 (15 pmol)		

[a] Same quantity of forward and reverse primers

Supplemental Table S3. Listing of antibodies tested as markers for CFTC isolation.

Marker	Reference	Supplier	Dilution	HTR-8	Trophoblasts[1]	Leukocytes[1]
CD105	130-094-941	Miltenyi	1/100	+	+	-
PanCK (8,18,19)	130-80-101	Miltenyi	1/100	+++	30% +	-
βhCG	NBP2-34632	Novus Biological	1/30	+	+	-
CK7	CBL194F	Millipore	1/50	-	+/-	-
EpCAM	130-080-30	Miltenyi	1/100	+/-	+/-	-
Vimentin	562337	BD Pharmingen	1/50	+++	+++	+++
HLAG	MA119610	Invitrogen	1/50	-	-	ND
EGFR	352908	Biolegend	1/50	+	+	-
E-cadherin	130-095-412	Miltenyi	1/100	-	ND	-
N-cadherin	350805	Biolegend	1/50	-	+	-
CD141	130-113-317	Miltenyi	1/100	-	-	ND
CD133	130-090-853	Miltenyi	1/100	-	ND	-
CD34	IM1870	Beckman Coulter	1/50	-	ND	-
CD44	IM1219	Beckman Coulter	1/50	++	++	++
CD24	130-095-953	Miltenyi	1/100	-	ND	-
CD146	130-092-849	Miltenyi	1/100	-	-	-
CD45	130-113-114	Miltenyi	1/100	-	-	+++
Her-2neu	ab106674	Abcam	1/100	+	+	-

[1] ND: Not determined

Supplemental Table S4. Classification criteria of each isolated cell according to their microsatellite profile

Categories	Criteria
Uninterpretable cell	no allele<br>or <2 parental alleles (not paternal-specific)<br>or >1 exogenous allele<br>or presence of 1 specific-paternal allele AND 2 maternal-specific alleles for one fully informative marker
Maternal cell	2 maternal-specific alleles for one fully informative marker
Inconclusive cell	>2 parental alleles (not paternal-specific)
Fetal cell	one or more paternal specific allele(s)

Supplemental Table S5. Families' characteristics and results of single-cell testing using the TruSight One gene panel.

Family	H	I	J	K	L	M	N	O	P
**Weeks of gestation+day**	12+1	12+1	13+4	15+6	12+1	10+6	11+6	12+1	13+2
**Gene**	*CFTR*	*NF1*	*MVK*	*SPINK5*	*CFTR*	*IGHMBP2*	*FMN2*	*RPGR*	*SEPSECS*
**Monogenic disease**	Cystic fibrosis	Neurofibromatosis type 1	Mevalonic aciduria	Netherton syndrome	Cystic fibrosis	Neuropathy, distal hereditary motor, type VI	Mental retardation, autosomal recessive 47	Cone-rod dystrophy, X-linked, 1	Pontocerebellar hypoplasia type 2D
**Reference sequence**	NM_000492.3	NM_000267.3	NM_000431 .1	NM_001127698.1	NM_000492.3	NM_02180.2	NM_001305424.1	NM_001034853.1	NM_016955.3
**Maternal mutation**	c.3909C>G, p.Asn1303Lys	c.288+3A>T	c.709A>T, p.Thr237Ser (exon 8)	c.153delT, p. Gln52Lysfs*6	c.1521_1523delCTT, p.Phe508del	c.1540G>A, p.Glu514Lys	c.4720C>T, p.Gln1574*	c.3039_3040delGG, p.Glu1014Glyfs*64	c.808dupG, p.Ala270Glyfs*5
**Paternal mutation**	c.1521_1523delCTT, p.Phe508del	none	c.1129G>A, p.Val377Ile (exon 11)	c.153delT, p. Gln52Lysfs*6	c.3909C>G, p.Asn1303Lys	c.2762G>C, p.Cys921Ser	c.4720C>T, p.Gln1574*	none	c.114+3A>G, p.Gly39Valfs*63
**Uninterpretable**	0	0	1	15	2	20	5	3	0
**Maternal**	0	0	0	0	0	1	0	0	0
**Inconclusive (maternal or fetal)**	0	0	0	1	0	8	0	0	0
**Fetal**	0	0	1	0	0	1	0	0	0
**Mini-exome coverage[1]**	n/a	n/a	23%	n/a	n/a	4%	n/a	n/a	n/a
**NIPD result[1]**	n/a	n/a	no	n/a	n/a	no	n/a	n/a	n/a

[1] n/a: not available

Supplemental Figures

Supplemental Figure S1. Localization of the microsatellites studied in the Huntington's disease multiplex PCR protocol

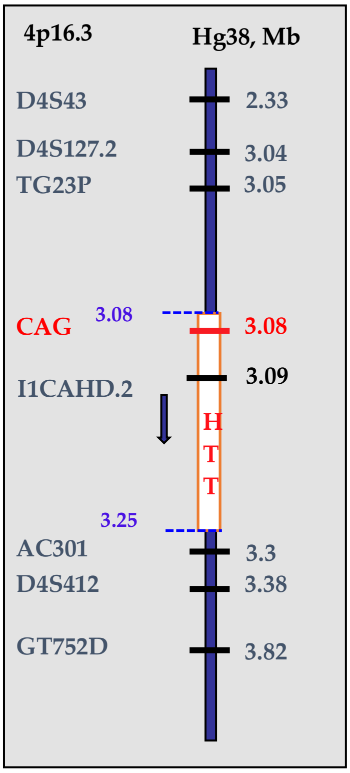

Supplemental Figure S2. Different steps of the methods tested for enrichment, detection and isolation of single CFTCs.

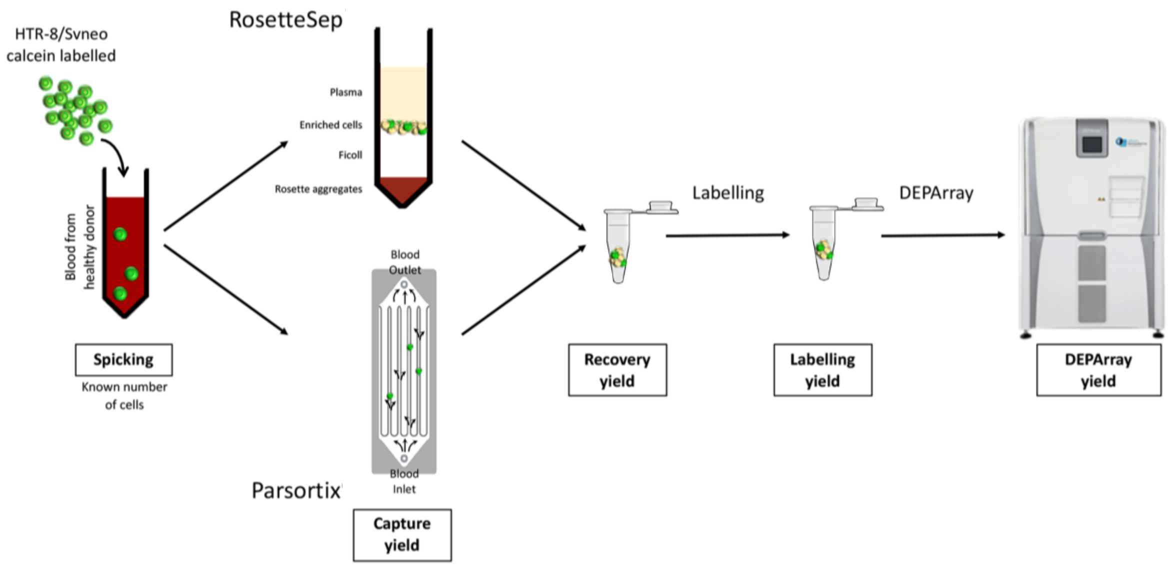

Supplemental Figure S3. Genotype profile of HTR-8/SVneo single cell obtained using the Huntington's disease multiplex PCR protocol.

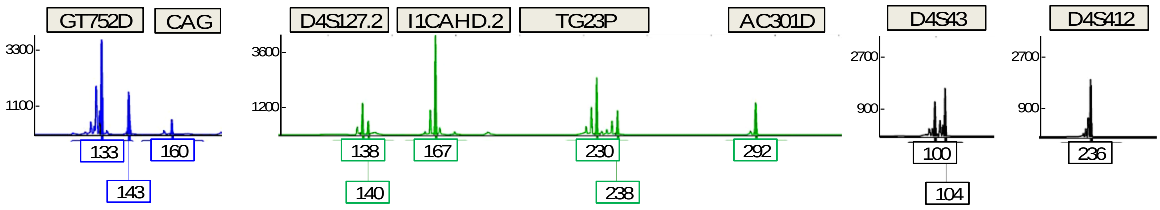

Supplement: Supplementary file 1 — Supplementary information. [file 41598_2020_66923_MOESM1_ESM.pdf]
